# Pheochromocytoma, paragangliomas, and pituitary adenoma: An unusual association in a patient with an SDHD mutation. Case report

**DOI:** 10.1097/MD.0000000000016594

**Published:** 2019-07-26

**Authors:** Annie Lemelin, Marion Lapoirie, Juliette Abeillon, Hélène Lasolle, Sophie Giraud, Pierre Philouze, Philippe Ceruse, Gérald Raverot, Alain Vighetto, Françoise Borson-Chazot

**Affiliations:** a Department of Endocrinology; b Department of Endocrinology, Hospices Civils de Lyon, Fédération d’Endocrinologie; c Department of Genetics; d Department of Oto-rhino-laryngology; e Department of Neurology, Hospices Civils de Lyon, Lyon 1 University, Lyon, France.

**Keywords:** paraganglioma, pheochromocytoma, pituitary adenoma, succinate dehydrogenase

## Abstract

**Rationale:**

Pituitary adenomas and paragangliomas are both rare endocrine diseases. Paragangliomas (PGL)/pheochromocytomas (PHEO) are part of an inherited syndrome in about 30% to 40% of cases. Among familial cases, mutations of the succinate dehydrogenase (SDH) subunit genes (succinate dehydrogenase subunit [SDH]B, SDHC, SDHD, succinate dehydrogenase subunit AF2 [SDHAF2] , and SDHA) are the most common cause.^[1]^

**Patient concerns:**

We here report a 31-year-old patient with a known SDHD mutation whose disease has been revealed by a left PHEO during childhood and who presented at age 29 years a large paraganglioma of the right jugular foramen, a concomitant PHEO of the left adrenal and 2 retroperitoneal paragangliomas. A pituitary incidentaloma was found during investigations on a fluorodeoxyglucose (FDG)-positron emission tomography (PET) (FDG-PET).

**Diagnosis:**

A pituitary magnetic resonance imaging (MRI) confirmed the presence of a 14 mm pituitary macroadenoma. The pituitary function was normal except for hypogonadotropic hypogonadism. On examination of the fundus, a diagnosis of Pseudo Foster-Kennedy syndrome was made due to a venous compression of the right jugular vein caused by the paraganglioma (PGL). The pituitary adenoma was not compressive to the optic chiasm.

**Interventions:**

A treatment with acetazolamide was started in order to improve intracranial hypertension. The patient couldn’t benefit of a surgical approach for the paraganglioma of the right jugular foramen; the patient has been treated with stereotactic radiosurgery (Gamma Knife).

**Outcomes:**

The most recent MRI revealed that the right jugular foramen PGL is stable and the latest visual assessment demonstrated stability despite a recent reduction in acetazolamide dosage. A surveillance by MRI of the pituitary adenoma has been planned.

**Lessons:**

The association of a pituitary adenoma to paragangliomas within a same patient is very uncommon and raises the question of related physiopathological mechanisms.

## 1 Introduction

Paragangliomas and pheochromocytomas (PPGL) are rare neuroendocrine tumors. Paragangliomas (PGL) originate from the extra-adrenal chromaffin cells of the sympathetic and parasympathetic system, whereas pheochromocytomas (PHEO) derive from chromaffin cells of the adrenal medulla.^[2]^ PHEOs and abdominal PGLs originating from the sympathetic system can secrete catecholamines, while cervical PGLs developed from the parasympathetic system are usually nonfunctional. PPGLs can be sporadic or familial. Up to 40% of cases may be associated with an inherited familial syndrome, including succinate dehydrogenase (SDH) enzyme mutations.^[3]^ Any mutation of the SDH complex subunit (SDHB, SDHC, SDHD, SDHAF2, and SDHA) predisposes mainly to the development of PPGL, but the recent literature also reported other SDH-related lesions such as gastrointestinal stromal tumors (GIST), renal cell carcinomas, and pituitary adenomas (PA).^[4,5]^ We describe here the case of a 31-year-old patient who presented the uncommon association of paragangliomas and pituitary adenoma. Informed consent was obtained from the patient for the purpose of publication.

## 2 Case presentation

**Figure 1:**
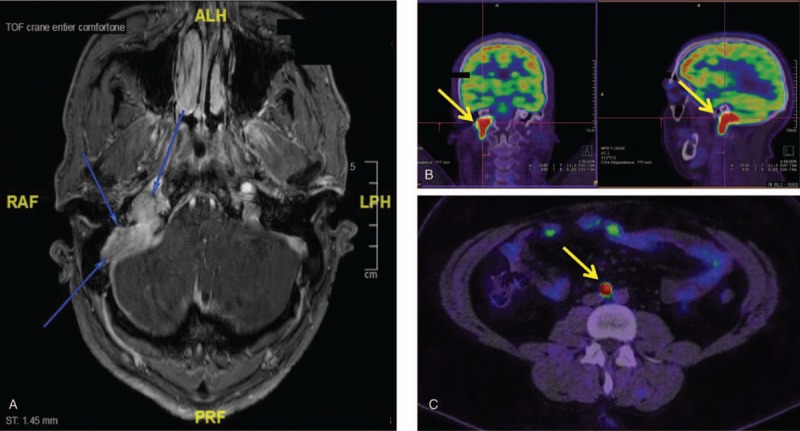
A: MRI showed a large PGL at the right posterior jugular foramen. B: High uptake on FDG-PET revealing a large PGL at the right posterior jugular foramen. C: FDG-PET: High uptake of an inter-aortico-cave lesion, compatible with a PGL. MRI = magnetic resonance imaging, PGL = paraganglioma.

A 31-year-old patient with a known succinate dehydrogenase enzyme complex D (SDHD) mutation underwent a left adrenalectomy for an adrenal pheochromocytoma (PHEO) at the age of 10, which was revealed by hypertensive peaks. He had a positive family history of SDHD mutation (exon 4, c.315-?_480+?del) inherited from his father and his two brothers also carry this mutation. He had a follow-up for a few years after surgery, but was unfortunately lost to follow up afterwards. He recently resumed a follow-up in the context of a recurrent hypertension documented following a car accident for which further investigations revealed a left adrenal nodule of 11 mm associated with 2 retroperitoneal lymph nodes. A cervical MRI was also performed and showed a large paraganglioma (PGL) at the right posterior jugular foramen of 41 × 44 × 12 mm (Fig. 1A). The patient was completely asymptomatic except for a bilateral tinnitus since several years. The plasma fractionated metanephrines were normal. An fluorodeoxyglucose (FDG)-positron emission tomography (PET) (FDG-PET) was performed and showed a hypermetabolism of all the lesions, compatible with a left adrenal PHEO and multiple PGLs in the context of the known SDHD mutation (Fig. 1B and C).

**Figure 2:**
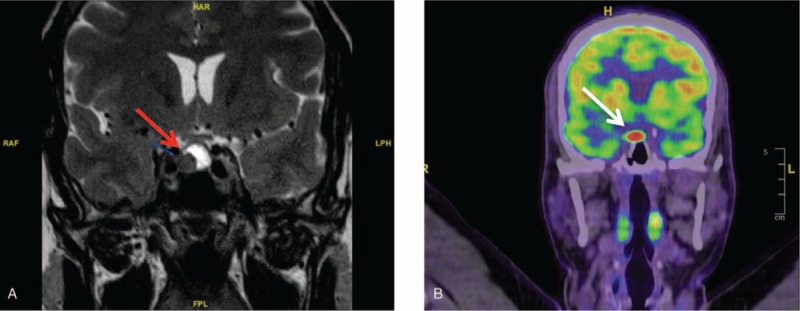
A and B: Pituitary adenoma discovered incidentally on PET-FDG and confirmed at MRI. MRI = magnetic resonance imaging.

Furthermore, a hypermetabolic pituitary lesion was found at FDG-PET and a pituitary MRI confirmed the presence of a 14 mm pituitary macroadenoma (PA) (Fig. 2A and B). The pituitary function was normal except for hypogonadotropic hypogonadism: corticotropin (ACTH) stimulation test: cortisol (T0) 190 nmol/L, (T60) 759 nmol/L (N > 374 nmol/L at T60), insulin-like growth factor-1 (IGF-1) 192 μg/L (N 98–282 μg/L), thyroid-stimulating hormone (TSH): 1.7 mU/L (N 0.4–3.1 mU/L), free thyroxine (T4L): 14.2 pmol/L (N 12.0–22.0 pmol/L), free triiodothyronine (T3L) 5.2 pmol/L (3.4–5.2 pmol/L), luteinizing hormone (LH): 1.26 UI/L (N 1.3–5.8 UI/L), follicle-stimulating hormone (FSH): 2.6 UI/L (N 1.1–7.2 UI/L), testosterone: 5.5 nmol/L (N 10.4–26 nmol/L), prolactin (PRL): 15.2 μg/L (N 4.0–15.2 μg/L). PA was nonsecreting and hypogonadism was attributed to PA in the context of a macroadenoma.

On examination of the fundus, Frisen Grade IV papilloedema was found in the right eye and an optic atrophy was noticed in the left eye. A lumbar puncture confirmed an intracranial hypertension of chronic evolution with an opening pressure of the cerebrospinal fluid at 31 cm H_2_O documented during the procedure (N ≤ 15 cm H_2_O). Visual acuity remained normal, but visual field abnormalities were found bilaterally. An enlargement of the blind spot and an amputation of the inferotemporal visual field on the right were noticed. On the left eye, there was a diffuse alteration of the visual field, but without enlarging of the blind spot. The patient was evaluated in neuro-ophthalmology and a diagnosis of Pseudo Foster-Kennedy syndrome was made. Additional investigations were performed in order to explore the causes of the intracranial hypertension. As a result, cerebral venous thrombosis was excluded and there was no expansive lesion directly compressing the optic nerves. Moreover, the PA was not compressive to the optic chiasm. However, a slowing of the venous flow in the right lateral sinus was suggested by the neuro-ophthalmologist at the PGL site. The final hypothesis retained for the cause of Pseudo Foster Kennedy syndrome was an intracranial hypertension due to a venous compression of the right jugular vein caused by the PGL. A treatment with acetazolamide was started in order to improve intracranial hypertension, which stabilized visual field alterations.

Regarding the management of the left adrenal PHEO and the 2 retroperitoneal PGL, it was decided in tumor board to opt for a clinical monitoring because of the non-secretory and asymptomatic nature of these lesions and to prioritize the treatment of the right jugular foramen PGL responsible of the intracranial hypertension. Unfortunately, the patient couldn’t benefit of a surgical approach; a too high surgical risk and morbidity associated with the procedure having contraindicated this approach. The option of radiotherapy was selected and the patient has been recently treated with stereotactic radiosurgery (Gamma Knife). A surveillance by MRI of the pituitary adenoma has been planned. The most recent MRI revealed that the right jugular foramen PGL is stable and the latest visual assessment demonstrated stability despite a recent reduction in acetazolamide dosage.

## 3 Discussion

The presence of PGL, PHEO, and PA within the same patient remains a rare association. The overall prevalence of pituitary incidentaloma is estimated up to 16% in autopsy and radiologic studies.^[6]^ PA is a benign tumor and is one of the common causes of sellar mass. PPGL are much rarer neoplasms and annual incidence is estimated to be 0.8 per 100,000 person years.^[7]^ Current data suggest that a hereditary syndrome is found in about 30% to 40% of the cases, which raises the critical importance of genetic testing in these patients.^[3,8]^ Mutation of the SDH subunit genes A, B, C, or D is a known cause of hereditary PGL with an autosomal dominant inheritance pattern. SDH is a tumor-suppressor gene and any mutation in SDH subunits predisposes to PPGL. SDHD mutation is the most frequent familial PGL syndrome and is an imprinted gene, PPGLs occurring mainly in patients who inherited the mutation from their father. SDHD mutation causes predominantly neck and skull base PGL, often multifocal and usually benign. The occurrence of PHEO is less frequent.^[8]^

Some cases of PA associated to PPGL have been reported in the literature. The first one, a patient with acromegaly and PHEO has been described in 1952.^[9]^ The coexistence of PA and SDHD mutation has been reported for the first time by Xekouki et al in 2012. They demonstrated a loss of heterozygosity at SDHD locus and a reduced SDHD protein expression in the pituitary adenoma in a patient with a somatotropic macroadenoma and a known SDHD mutation.^[10]^ In addition, O’Toole et al reported in 2015 a compilation of 72 patients with concomitant PA and PPGL from whom only 5 had a SDHD mutation.^[5,10–12]^ All PA associated with SDHD mutation were functional macroadenomas.^[13]^ Besides, even though the data remains little known, some authors hypothesize that PAs associated with SDH mutations might behave more aggressively and be more resistant to treatment.^[5,14]^ In addition, it is suggested that these PAs could be more frequently prolactinomas and somatotropic adenomas. In our case, the PA was not secretant and whether SDHD was expressed or mutated within the tumor unknown since the patient has not been operated.

Infirst reports, it was thought that the combination of PA and PPGL was a simple coincidence, but recent data suggest a possible association. Interestingly, Gill et al in 2014 studied the incidence of SDH mutation in 309 PA by immunohistochemistry for SDHA and SDHB. They found only 1 PA (0.3%) with an abnormal pattern of staining; this patient had no germline SDH mutation. They concluded that SDH mutation is a very rare event in an unselected population with PA.^[15]^ Moreover, in 2015, Xekouki et al studied the prevalence of SDHx mutation in a cohort of 168 patients with unselected PA. They sought for the association of PA and PPGL; a condition they named 3P association (3PAs) and related SDHx germline mutation. Overall, only 1.8% of case of PA showed an SDHx mutation. Among 146 sporadic cases of PA, they found 3 cases of 3PAs association and genetic analysis showed no SDH mutation in these patients. Whereas in 22 familial cases of PA, 4 cases of 3PAs association were identified and 75% harbored an SDH germline mutation. They suggested that SDH mutation-associated PAs was more common among familial cases. They also studied the evolution of the pituitary gland in SDHB mutated mice and reported that SDHB mutated mice had more pituitary hyperplasia and a higher proportion of prolactin and IGF-1 producing cells, suggesting a possible association between PA and PPGL, probably related to SDH germline mutation.^[5]^ Furthermore, Dénes et al from a series of 39 patients with sporadic or familial PA and PPGL described 8 cases of mutation of the SDH complex and both PA and PPGL.^[16]^ Besides, this series revealed a particular histologic feature in all patients with PA and an SDH mutation. They found intracytoplasmic vacuoles in the pituitary tumor, a characteristic that could be a unique feature in these patients with concomitant PA, PPGL, and SDH mutation. Taken together, these data suggest that the association may not be fortuitous. However, these data need to be confirmed with further research.

In conclusion, these data suggest that the association between PA and PPGL could be more than just a coincidence in our patient, the existence of a predisposition to PA in patients with PPGL and SDHD mutation might be involved, but this finding needs further investigation.

## Author contributions

**Methodology:** Françoise Borson-Chazot.

**Supervision:** Françoise Borson-Chazot.

**Validation:** Françoise Borson-Chazot.

**Writing – original draft:** Annie Lemelin, Gérald Raverot, Françoise Borson-Chazot.

**Writing – review & editing:** Marion Lapoirie, Juliette Abeillon, Hélène Lasolle, Sophie Giraud, Pierre Philouze, Philippe Ceruse, Gérald Raverot, Alain Vighetto, Françoise Borson-Chazot.

## References

[1] Burnichon N Rohmer V Amar L The succinate dehydrogenase genetic testing in a large prospective series of patients with paragangliomas. J Clin Endocrinol Metab 2009;94:2817–27. 19454582 10.1210/jc.2008-2504

[2] DeLellis RA Lloyd R Heitz PU Eng C Pathology and Genetics of Tumours of the Endocrine Organs. WHO Classification of Tumours. 2004;France: IARC press, 8.

[3] Fishbein L Merrill S Fraker DL Inherited mutations in pheochromocytoma and paraganglioma: why all patients should be offered genetic testing. Ann Surg Oncol 2013;20:1444–50. 23512077 10.1245/s10434-013-2942-5 PMC4291281

[4] Mannelli M Canu L Ercolino T DIAGNOSIS of ENDOCRINE DISEASE: SDHx mutations: beyond pheochromocytomas and paragangliomas. Eur J Endocrinol 2018;178:R11–7. 28924001 10.1530/EJE-17-0523

[5] Xekouki P Szarek E Bullova P Pituitary adenoma with paraganglioma/pheochromocytoma (3PAs) and succinate dehydrogenase defects in humans and mice. J Clin Endocrinol Metab 2015;100:E710–9. 25695889 10.1210/jc.2014-4297 PMC4422891

[6] Ezzat S Asa SL Couldwell WT The prevalence of pituitary adenomas: a systematic review. Cancer 2004;101:613–9. 15274075 10.1002/cncr.20412

[7] Beard CM Sheps SG Kurland LT Occurrence of pheochromocytoma in Rochester, Minnesota, 1950 through 1979. Mayo Clin Proc 1983;58:802–4. 6645626

[8] Welander J Soderkvist P Gimm O Genetics and clinical characteristics of hereditary pheochromocytomas and paragangliomas. Endocr Relat Cancer 2011;18:R253–76. 22041710 10.1530/ERC-11-0170

[9] Iversen K Acromegaly associated with phaeochromocytoma. Acta Med Scand 1952;142:1–5. 14923268 10.1111/j.0954-6820.1952.tb13837.x

[10] Xekouki P Pacak K Almeida M Succinate dehydrogenase (SDH) D subunit (SDHD) inactivation in a growth-hormone-producing pituitary tumor: a new association for SDH? J Clin Endocrinol Metab 2012;97:E357–66. 22170724 10.1210/jc.2011-1179 PMC3319210

[11] Varsavsky M Sebastian-Ochoa A Torres Vela E Coexistence of a pituitary macroadenoma and multicentric paraganglioma: a strange coincidence. Endocrinol Nutr 2013;60:154–6. 22575350 10.1016/j.endonu.2012.02.009

[12] Papathomas TG Gaal J Corssmit EP Non-pheochromocytoma (PCC)/paraganglioma (PGL) tumors in patients with succinate dehydrogenase-related PCC-PGL syndromes: a clinicopathological and molecular analysis. Eur J Endocrinol 2014;170:1–2. 24096523 10.1530/EJE-13-0623

[13] O’Toole SM Denes J Robledo M 15 YEARS OF PARAGANGLIOMA: The association of pituitary adenomas and phaeochromocytomas or paragangliomas. Endocr Relat Cancer 2015;22:T105–22. 26113600 10.1530/ERC-15-0241

[14] Benn DE Robinson BG Clifton-Bligh RJ 15 YEARS OF PARAGANGLIOMA: clinical manifestations of paraganglioma syndromes types 1-5. Endocr Relat Cancer 2015;22:T91–103. 26273102 10.1530/ERC-15-0268 PMC4532956

[15] Gill AJ Toon CW Clarkson A Succinate dehydrogenase deficiency is rare in pituitary adenomas. Am J Surg Pathol 2014;38:560–6. 24625421 10.1097/PAS.0000000000000149 PMC3966922

[16] Denes J Swords F Rattenberry E Heterogeneous genetic background of the association of pheochromocytoma/paraganglioma and pituitary adenoma: results from a large patient cohort. J Clin Endocrinol Metab 2015;100:E531–41. 25494863 10.1210/jc.2014-3399 PMC4333031

